# Development of an Effective and Stable Genotype-Matched Live Attenuated Newcastle Disease Virus Vaccine Based on a Novel Naturally Recombinant Malaysian Isolate Using Reverse Genetics

**DOI:** 10.3390/vaccines8020270

**Published:** 2020-06-02

**Authors:** Muhammad Bashir Bello, Siti Nor Azizah Mahamud, Khatijah Yusoff, Aini Ideris, Mohd Hair-Bejo, Ben P. H. Peeters, Abdul Rahman Omar

**Affiliations:** 1 Laboratory of Vaccines and Immunotherapeutics, Institute of Bioscience, University Putra Malaysia, Serdang, Selangor 43400, Malaysia; bbtambuwal@gmail.com (M.B.B.); snazizahmahamud@gmail.com (S.N.A.M.); kyusoff@upm.edu.my (K.Y.); aiini@upm.edu.my (A.I.); mdhair@upm.edu.my (M.H.-B.); 2 Department of Veterinary Microbiology, Faculty of Veterinary Medicine, Usmanu Danfodiyo University PMB 2346 Sokoto, Nigeria; 3 Center for Advanced Medical Research and Training, Usmanu Danfodiyo University, PMB 2346 Sokoto, Nigeria; 4 Department of Microbiology, Faculty of Biotechnology and Biomolecular Sciences, Universiti Putra Malaysia, Serdang, Selangor 43400, Malaysia; 5 Department of Veterinary Clinical Studies, Faculty of Veterinary Medicine, University Putra Malaysia, Serdang, Selangor 43400, Malaysia; 6 Department of Veterinary Pathology and Microbiology, Faculty of Veterinary Medicine, University Putra Malaysia, Serdang, Selangor 43400, Malaysia; 7 Department of Virology, Wageningen Bioveterinary Research, POB 65, NL8200 Lelystad, The Netherlands; ben.peeters@wur.nl

**Keywords:** Newcastle disease virus, reverse genetics, recombinant vaccine, genotype-matched, genotype VII

## Abstract

Genotype VII Newcastle disease viruses are associated with huge economic losses in the global poultry industry. Despite the intensive applications of vaccines, disease outbreaks caused by those viruses continue to occur frequently even among the vaccinated poultry farms. An important factor in the suboptimal protective efficacy of the current vaccines is the genetic mismatch between the prevalent strains and the vaccine strains. Therefore, in the present study, an effective and stable genotype-matched live attenuated Newcastle disease virus (NDV) vaccine was developed using reverse genetics, based on a recently isolated virulent naturally recombinant NDV IBS025/13 Malaysian strain. First of all, the sequence encoding the fusion protein (F) cleavage site of the virus was modified in silico from virulent polybasic (RRQKRF) to avirulent monobasic (GRQGRL) motif. The entire modified sequence was then chemically synthesized and inserted into pOLTV5 transcription vector for virus rescue. A recombinant virus termed mIBS025 was successfully recovered and shown to be highly attenuated based on OIE recommended pathogenicity assessment indices. Furthermore, the virus was shown to remain stably attenuated and retain the avirulent monobasic F cleavage site after 15 consecutive passages in specific-pathogen-free embryonated eggs and 12 passages in one-day-old chicks. More so, the recombinant virus induced a significantly higher hemagglutination inhibition antibody titre than LaSota although both vaccines fully protected chicken against genotype VII NDV induced mortality and morbidity. Finally, mIBS025 was shown to significantly reduce both the duration and quantity of cloacal and oropharyngeal shedding of the challenged genotype VII virus compared to the LaSota vaccine. These findings collectively indicate that mIBS025 provides a better protective efficacy than LaSota and therefore can be used as a promising vaccine candidate against genotype VII NDV strains.

## 1. Introduction

Newcastle disease is one of the most important avian diseases with devastating economic consequences in the global poultry industry [1]. Although the disease affects a wide range of wild and domestic avian species, it is particularly more important in domestic chicken where it is manifested in different clinical forms involving gastrointestinal, neurological and respiratory systems [2,3]. Following its first official documentation about 100 years ago in England, the disease has continued to threaten poultry production and negatively impact on food security in different parts of the world [4,5]. Given its tendency to rapidly deplete poultry resources during outbreaks, ND has been included in the list of diseases that must be reported to the Organization of International Epizootics (OIE) immediately upon recognition [6]. The disease is caused by Newcastle disease virus (NDV), a member of *Paramyxoviridae* family in the genus avian avulavirus-1. The genome of the virus is a negative-stranded, non-segmented RNA of about 15.2 kb in size [7] which is made up of six genes encoding nucleoprotein (NP), phosphoprotein (P), matrix protein (M), fusion protein (F), hemagglutinatinin-neuraminidase protein (HN) and large protein (L) [8]. In addition to these structural proteins, two other non-structural proteins, V and W, are also produced by RNA editing of the P gene during transcription [9,10].

The most important protein in NDV virulence is the F protein [11]. Under normal circumstances, it is synthesized in an inactive form F_0_, but cleaved enzymatically into F1 and F2 in order to activate its full biological functions during infection [12]. Interestingly, the amino acid composition of the F cleavage site can be used to categorize NDV into virulent and non-virulent isolates. According to the OIE, virulent NDV isolates are identified by the possession of multiple basic amino acids (arginine and lysine) at positions 112–116, and a phenylalanine at position 117 of the F protein [13]. This polybasic amino acid composition is susceptible to the proteolytic activity of the ubiquitously distributed furin-like proteases, explaining the widespread multi-systemic pathology of the virulent NDV isolates. On the other hand, isolates with monobasic F cleavage site at the carboxy terminus of F2 and a leucine residue at position 117 are generally considered to be strains of low virulence with restricted tissue pathology because their F protein is only cleavable by extracellular trypsin-like proteases only found in the digestive and respiratory systems. Thus, the amino acid composition of the F cleavage site is used as a good indicator of NDV virulence [14]. Other OIE recommended indicators of NDV virulence include the mean death time (MDT) performed in 9- to 10 day-old embryonated chicken eggs and the intracerebral pathogenicity index (ICPI) performed in one-day-old chicks. Both tests are used to classify NDV isolates into lentogenic (mildly virulent), mesogenic (moderately virulent) and velogenic (highly virulent) strains [15,16] even though ICPI is generally more preferred as an official and international NDV pathogenicity assessment test.

Although all NDV strains are classified under one serotype, their genetic diversity is enormous. Previously, two major schemes were used in the molecular classification of NDV. The first scheme classifies the NDV isolates into lineages (I–VI) and their respective sublineages [17]. The other classification scheme proposed by Ballagi-Pordány et al. [18,19], divides the NDV isolates broadly into class I and class II with several genotypes and subgenotypes in each class. Notably, with the exception of a few isolates, all class I members uniquely have a genome length of 15,198 bp and are distributed worldwide in wild birds. They are also usually avirulent in chicken [20]. On the other hand, the class II viruses constitute both the virulent and avirulent strains and their total genome length is usually 15,186 bp for genotypes isolated before 1960 (early genotypes) or 15,192 bp in the case of late genotypes that were isolated after 1960 [19]. Interestingly, a more comprehensive criteria for NDV classification was recently adopted in order to bring an end to the confusion created by simultaneous usage of the two schemes of NDV taxonomy [21]. Based on this unified system of classification, NDV isolates are grouped into class I, with only one genotype and class II having up to 18 genotypes [22]. Importantly, all members of class II genotypes I and II, with the exception of a neurotropic virulent chicken strain, isolated in 1948 in the United States, are of low virulence in chicken [23]. Indeed most of the popular commercially available ND vaccines such as LaSota and Hitchner B1 are derived from these genotype II isolates [24,25]. Genotypes III to XVIII are however mostly composed of strains that are highly pathogenic in chicken [24]. More recently, a more stringent NDV nomenclature system proposed by an international consortium of experts increased the number of genotypes from 18 to 21, although the total number of subgenotypes has reduced [26].

Over the last twenty years, the sustainable growth of the poultry industry has considerably been suppressed by the numerous disease outbreaks caused by genotype VII NDV isolates [27,28,29]. These isolates are believed to be responsible for the on-going fourth, and the imminent fifth ND panzootics [30] because of their rapid expansion of geographic distribution and frequent isolation from farms that have vaccinated using the conventional genotype II-based vaccines [31,32]. This continuous emergence of genotype VII NDV in vaccinated farms demonstrates the suboptimal efficacy of the current vaccines largely due to the phylogenetic divergence between the vaccine strains and the currently prevalent NDV isolates (Table 1). Thus, there is a need for improved vaccines in order to curtail the menace of those viruses. It is widely believed that the best strategy of addressing the menace of genotype VII NDV is the production of genotype-matched vaccines which have been shown to demonstrate higher protective efficacy than their genotype mismatched counterparts [31,33,34]. Therefore, in the present study, we report the use of reverse genetics to develop a highly effective, safe, and stable vaccine against the prevailing NDVs in Malaysia, based on the recently isolated naturally recombinant genotype VII isolate.

## 2. Materials and Methods

### 2.1. Cells and Plasmids

Baby hamster kidney (BHK-21) cells genetically engineered to constitutively express T7 polymerase (BSR-T7) were a kind gift from Prof. Conzelmann in Germany. The cells were maintained in GMEM (,Thermo Fisher Scientific, Waltham, MA USA) supplemented with 10% newborn calf serum, 1% tryptose phosphate broth, 2% MEM amino acids, and 1% Penicillin-streptomycin antibiotics. One (1) mg/mL G418 antibiotic (Sigma Aldrich, Germany) was used to select T7 RNA polymerase expressing cells during each passage of the cells. Transcription vector pOLTV5-phix is derived from pOLTV5. The vector contains a kanamycin resistance gene and has a T7 promoter and T7 terminator separated by a bacteriophage phiX174 stuffer sequence of about 620 bp flanked by *Bbs*I restriction sites. In addition, the T7 terminator is preceded by an HDV ribozyme sequence in the vector. pCIneo mammalian expression vector was purchased from Sigma-Aldrich(Germany).

### 2.2. Construction of Helper Plasmids

RNA was extracted from allantoic fluid infected with the wild type NDV strain IBS025/13 using Trizol LS^®^ reagent (Invitrogen, Carlsbad, USA) following the instructions of the manufacturer. To synthesize cDNA, 0.7–1µg of the extracted RNA was reverse transcribed using a Sensifast^®^ cDNA synthesis kit (Bioline, UK) according to the recommended protocol provided in the kit. The complete open reading frames of the viral NP, P, and L genes were amplified from the cDNA template and then subcloned into pCIneo to generate helper plasmids (pCIneo-NP, pCIneo-P and pCIneo-L). All cloning experiments were verified using colony PCR, restriction endonuclease digestion, and DNA sequencing. Purity and concentrations of each recombinant plasmid construct were determined using Nanodrop 2000 (Eppendorf, Germany).

### 2.3. In Vitro Expression of Helper Plasmids

To verify the ability of the helper plasmids to express the encoded proteins, indirect immunofluorescence assay (IFAT) was performed. Accordingly, 3µg of either pCIneo-NP or pCIneo-P was individually transfected into BHK-21 cells at 70–80% confluence in six well-cell culture plates using lipofectamine 2000 reagent (Invitrogen, USA). Negative control wells were transfected with empty pCIneo vector. After 72 h of incubation, the cells were fixed with 4% paraformaldehyde and permeabilized with 0.25% Triton X100. Next, 1% bovine serum albumin (BSA) was used to block non-specific binding sites before incubating the cells with chicken anti-NDV polyclonal antibody (ABCAM, UK) at 4 °C overnight. FITC-conjugated goat anti-chicken IgY (ABCAM, UK) was used as a secondary antibody. Finally, the cells were counter stained using SlowFade^®^ Gold antifade reagent with DAPI (Life Technologies, USA) for 10 min and immediately viewed under a fluorescence microscope.

### 2.4. Construction of Full Length Antigenomic Plasmid

Using the complete genome sequence of NDV IBS025/13 (GenBank ID KT355595.1) as a template, the region encoding the F protein cleavage site was modified in silico from polybasic to monobasic amino acids residues, consistent with classically attenuated NDV strain LaSota. To do this, five nucleotide substitutions were made at specific genomic locations: A-G (4877), AAA-GGG (4886, 4887, and 4888) and T-C (4892). These site-directed substitutions modified the putative F cleavage site from ^112^RRQKRF^117^ to ^112^GRQGRL^117^. The modified sequence named NDV mIBS025, was flanked with *Bsm*BI restriction sequences and then sent for chemical synthesis (Genscript, USA). The synthesized antigenome (15.2 kb) was then bluntly cloned into pUC57-Brick via *Eco*RV site and named pUC-mIBS025. After propagation in NEB10beta competent cells, the full-length antigenome was excised from pUC-mIBS025 using *Bsm*BI enzyme and then purified using a gel extraction kit (Qiagen, Germany). Similarly, pOLTV5-phix was digested with *Bbs*I to remove the phix region from the vector. Finally, purified mIBS025 antigenome was successfully subcloned into purified pOLTV5 via the *Bsm*BI/*Bbs*I sites to form pOLTV5-mIBS025 full-length antigenomic plasmid.

### 2.5. Recovery of the Recombinant NDV mIBS025

To recover the recombinant virus, actively dividing BHK-21 cells at 80% confluence were co-transfected with a cocktail of helper plasmids and the plasmid encoding the full-length mIBS025 cDNA (pOLTV5-mIBS025) using lipofectamine 3000 reagent (Life Technologies) according to manufacturer’s instructions. Briefly, 5µL of Lipofectamine 3000 was first diluted in 125 µL of Opti-MEM^®^ reduced serum medium at room temperature. Then, 500 ng of pCIneo-NP, 250 ng of pCIneo-P, 200 ng of pCIneo-L, and 1000 ng of pOLTV5-mIBS025 constructs were mixed together and diluted in another 125 µL of Opti-MEM^®^ containing 5 µL of p3000 buffer. Next, the diluted transfection reagent was mixed with the diluted plasmid DNA cocktails and later incubated for 30 min at room temperature for optimal lipid-DNA complex formation. The transfection mixture along with 2µg of acetylated trypsin was then gently added to each well followed by incubation at 37 °C in a CO_2_ incubator. Four days later, the transfected cells were freeze-thawed three times along with their supernatants and later filtered using a sterile 0.2 μm syringe filters. About 500 μL of the filtrate from each well was then inoculated into the allantoic cavity of 9 to 10-day-old SPF chicken embryonated egg and incubated at 37 °C for 96 h. Subsequently, allantoic fluid was aseptically harvested from the inoculated eggs and tested by a simple hemagglutination test to identify the rescued virus. Allantoic fluid with hemagglutination (HA) activity was further subjected to RNA extraction and RT-PCR using specific primers targeting a partial F gene region encompassing the F protein cleavage site. PCR amplicons were later sequenced to identify the genetic tags in the rescued virus.

### 2.6. Ethical Clearance

Animal handling procedures were performed in line with the national animal welfare regulations. All experiments including in vitro manipulation of the virus, pathogenicity, in vivo stability, vaccine efficacy and challenge studies were performed in a BSL-2 facility and were approved by the Institutional Animal Care and Use Committee (IACUC), Faculty of Veterinary Medicine, Universiti Putra Malaysia (reference number UPM/IACUC/AUP-R005/2017) and the Institutional Biosafety Committee (reference number JBK(S) 602-1/2/186).

### 2.7. Pathogenicity of the Rescued Virus

Following the successful rescue of NDV mIBS025, the virus was characterized for pathogenicity using standard assays. Accordingly, mean death time (MDT) in 10-day-old SPF eggs and intracerebral pathogenicity index (ICPI) in one-day-old SPF chicks were performed according to the recommendations of OIE.

### 2.8. Determination of F Protein Cleavage Site and Phenotype Stability

To determine the stability of NDV mIBS025 F cleavage site, the virus was passaged both in SPF chicken embryonated eggs and in one-day-old SPF chicks. To passage mIBS025 in SPF eggs, 0.1 mL of the virus at 7 log2 HA titer was inoculated into the allantoic cavity of five 10-day-old SPF chicken embryonated eggs and incubated at 37 °C. After 5 days of incubation, allantoic fluids were harvested and tested for hemagglutination activity before inoculation into another set of SPF eggs. This process was repeated for 15 consecutive times (P1-P15). RNA was extracted from the allantoic fluid of various passages (P1, P3, P5, P10, and P15) for RT-PCR and sequencing of the partial F gene to determine the stability of the monobasic F cleavage site. For passaging in one-day-old chicks, 0.2 mL of the virus at 7 log2 HA titer was inoculated via the occulo-nasal route into five one-day-old chicks. The inoculated chicks were monitored for 5 days after which they were humanely sacrificed to obtain the infected lungs and trachea. The collected organs were aseptically homogenized, filtered, centrifuged, and subsequently inoculated into another set of three SPF chicken via the same route up to 12 times. RNA was also extracted from the respiratory organs infected with the virus at each passage and then analyzed by RT-PCR to determine the genetic stability of the monobasic F cleavage site. For the determination of ICPI, virus obtained from egg passages (P1, P3, and P5) was evaluated using ICPI based on OIE recommended method to determine the stability of the lentogenic phenotype of the virus.

### 2.9. Vaccine Efficacy Trial

Seven-day-old SPF embryonated chicken eggs obtained from Malaysia Vaccines and Pharmaceuticals (MVP) were maintained and hatched in our laboratory under sterile conditions. The hatched chickens were transferred to the animal house facility at Biologics Laboratory, Universiti Putra Malaysia where they were housed in clean stainless steel birdcages and fed with pelleted chicken feed. Water was also provided ad libitum while the drinkers were washed on a daily basis. The chicks were randomly divided into three groups: I, II, and III each comprising 10 birds housed in separate rooms. Groups I and II comprised the vaccinated groups while the chickens in group III constituted the unvaccinated control group. Birds in each group were tagged prior to the commencement of the experiment to allow the accurate monitoring of the individualistic response to vaccine and challenge viruses. Prior to vaccination, 0.5 mL of blood samples were collected from each bird in each group using 1 mL insulin syringe via the heart, in order to check for pre-vaccination NDV antibody titer using hemagglutination inhibition (HI) test. Each bird in group I was then vaccinated via the occulo-nasal route with 0.05 mL containing 10^6^ EID_50_ of NDV mIBS025 while the birds in group II were vaccinated with 10^6^ EID_50_ of NDV LaSota (0.05 mL) via the same route and group III members were occulonasally given an equivalent amount of PBS. Birds were monitored for post-vaccinal respiratory reactions and were bled on days 7, 14, and 21 post-vaccination to determine the immunogenicity of the respective vaccines using HI assays.

### 2.10. Serological Testing

Serum samples collected at days 0, 7, 14, and 21 after vaccination in various groups were analyzed by HI test using NDV strains LaSota (genotype II) and IBS025/13 (genotype VII) as HA antigens. The HI test was performed as described by the OIE Manual of Diagnostic Tests and Vaccines for Terrestrial Animals (OIE, 2004). HI titers were recorded as the highest dilution of the serum that completely inhibited the agglutination of chicken erythrocytes by the HA antigens. All tests were performed in triplicates to ensure the accuracy of the results.

### 2.11. Chicken Trial

Twenty-one days after vaccination, all the birds were challenged with 10^5^.^5^ ELD_50_ NDV strain IBS002/11 via the occulo-nasal routes [31,33]. The challenge virus is a virulent genotype VII isolate obtained from a disease outbreak in a broiler farm in 2011 [31]. It is classified as velogenic based on its ICPI value of 1.76 and the presence of polybasic amino acid residue at its F cleavage site (^112^RRRKRF^117^) [35]. The challenged birds were monitored for clinical signs and mortality for a period of 14 days. During this period, mortality was monitored while morbidity was scored according to recommendations by [36]. Accordingly, birds were scored 0 when normal, 1 when the disease was mild, 2 if it was moderate and 3 when severe. Cloacal and oro-pharyngeal swabs were also collected from each bird at days 3, 5, 7, 10, and 12 post-challenge using sterile wooden swab sticks. The swabs were placed in 1 mL sterile PBS and transported on ice to the laboratory for further processing. When the samples were not processed immediately, they were stored at −80 °C until needed.

### 2.12. Virus Shedding Determination

Cloacal and oropharyngeal swabs collected from all groups at various time points post challenge were used to extract total RNA using RNeasy^®^ mini kit Plus (Qiagen, Germany). Known concentration of RNA extracted from allantoic fluid infected with NDV strain IBS002/11 was used to construct a standard curve. Subsequently, to estimate the viral load, iScript™ One-Step qPCR Kit (Bio-Rad, USA) for Probes containing 2x reaction buffer made up of 0.25 mM of each dNTP, magnesium ions and iTaq DNA polymerase, stabilizers as well as 1 μL of iScript reverse transcriptase was utilized. The primers and probes originally designed by Rasoli et al. [37] to detect velogenic Malaysian NDV isolates were adopted in this study. All RNA samples were adjusted to a concentration of 200 ng/3.3 μL before the reactions were run. The reaction components were mixed in 0.2 mL tube strips placed on ice in order to avoid primer dimerization. Accordingly, 0.2 μL of iScript reverse transcriptase, 0.5 μL of 20 μM forward primer, 0.5 μL of 20 μM reverse primer and 0.5 μL of 10 μM probe were mixed with 5 μL of iScript™ One-Step qPCR mix. To top up the reaction to 10 μL, 3.3 μL of the template was added. The tubes were briefly vortexed and gently centrifuged before placing them in CFX 96 qPCR system (Bio-Rad, USA). Thermocycler conditions were 50 °C for 10 min, 95 °C for 5 min and 40 cycles of 95 °C for 10 s, 58 °C for 30 s before plate reading at the end of the reaction.

### 2.13. Data Analysis

All data generated were organized into tables and figures using Microsoft Office Excel. Geometric mean titers plus or minus (±) the standard errors of mean were used to represent HI titers and virus shedding results. Repeated measures analysis of variance (ANOVA) was used to analyze significant differences between the vaccinated groups and unvaccinated control group. Values of *p* < 0.05 were considered significant.

## 3. Results

### 3.1. Construction of Functional Helper Plasmids

**Figure 1:**
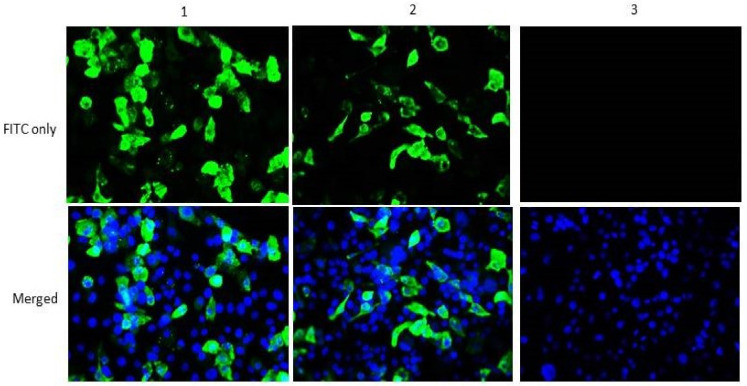
Immunofluorescence based detection of nucleoprotein (NP) and phosphoprotein (P) encoded by the helper plasmids. Actively dividing Baby hamster kidney (BHK-21) cells at 80% confluence were transfected with (**1**) pCIneo-NP. (**2**) pCIneo-P and (**3**) empty pCIneo plasmid (negative control). At 72 h post-transfection, the cells were fixed, permeabilized, and stained with chicken polyclonal IgY against Newcastle Disease Virus (NDV) followed by goat anti-chicken antibody conjugated with FITC. The cells were counter-stained with DAPI. Green and blue colors respectively represent FITC and DAPI. Images were captured using Zenlite fluorescence imaging software at X 200.

The open reading frames of NP, P, and L genes of NDV mIBS025/13 were amplified by RT-PCR and directionally cloned into pCIneo mammalian expression vector. The orientation of all the inserts was verified using colony PCR and restriction digestion (Figure S1). Sequencing of positive clones revealed a complete similarity with the reference virus sequence. In order to examine the ability of the helper plasmids to express the encoded proteins, NP and P constructs were used to transiently transfect BHK-21 cells and later analyzed by IFAT using chicken anti-NDV polyclonal antibody (primary antibody) and FITC conjugated goat anti-chicken antibody (ABCAM, UK). As expected, fluorescence was observed only in the wells transfected with pCIneo-NP or pCIneo-P but not in those transfected with empty pCIneo vector (negative control) (Figure 1). This shows that the helper plasmids encode proteins that are readily expressed and specifically identified by chicken anti-NDV polyclonal antibody.

### 3.2. Rescue of Recombinant NDVmIBS025

**Figure 2:**
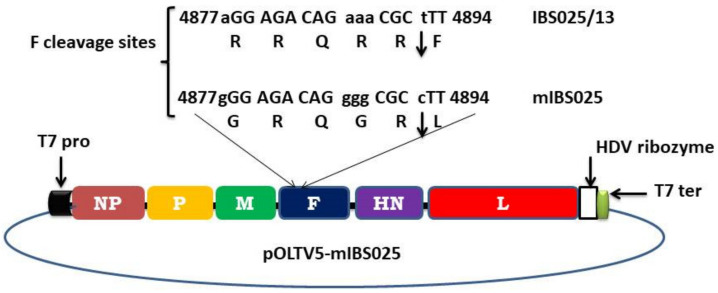
Construction of full length antigenomic clone of NDV mIBS025. Complete genome of the wild type NDV IBS025/13 was modified in silico at the fusion (F) protein cleavage site (4877–4894 bp) to generate monobasic amino acid residues at the site. Substituted nucleotides are shown in lower cases and the location of the cleavage is indicated by an arrow between the amino acids. The modified antigenome (NDV mIBS025) was synthesized and cloned in pOLTV5 transcription vector in between T7 promoter and autocatalytic hepatitis delta virus ribozyme sequence to produce pOLTV5-mIBS025 full length antigenomic construct.

To construct and rescue the lentogenic counterpart of NDV IBS025/13 (NDV mIBS025), nucleotide sequence modification aimed at changing the putative cleavage site motif from ^112^RRQKRF^117^ to ^112^GRQGRL^117^ were made in silico. The modified antigenome was then entirely synthesized and successfully subcloned into pOLTV5 transcription vector in between the T7 promoter and autocatalytic HDV ribozyme (Figure 2). Sequencing of the whole construct revealed a complete similarity with the in silico modified antigenome sequence. 

To rescue the recombinant virus, helper plasmids and the full lenght antigenomic construct were co-transfected into BHK-21 cells. When the transfection supernatants were inoculated into SPF embryonated eggs, recombinant virus with HA titers ranging from 7 log2 to 9 log2 was recovered. Further analysis of the HA positive allantoic fluid using RT-PCR and DNA sequencing revealed the presence of a monobasic F cleavage site consistent with all lentogenic NDV. This indicates the successful recovery of the NDV mIBS025.

### 3.3. Pathogenicity of the Recombinant Virus

Mean death time (MDT) and ICPI were used to evaluate the pathogenicity of recombinant and wild type viruses as per the prescription of the OIE. According to the OIE, NDV isolates are classified as velogenic, mesogenic, and lentogenic if their MDT values are less than 60 h, 60–90 h, and above 90 h respectively. On the other hand, OIE recommends ICPI values of 0.00–0.7 for lentogenic, 0.7–1.3 for mesogenic, and 1.3–2.0 for velogenic NDV. As shown in Table 2, the MDT values for wild type NDV IBS025/13 and the rescued NDV mIBS025 were < 60 h and > 120 h, respectively, while their ICPI values were 1.86 and 0.00, respectively. This indicates that the rescued NDV mIBS025 is lentogenic while the wild type NDV IBS025/13 is highly virulent.

### 3.4. F Protein Cleavage Site and Phenotypic Stability of the Recombinant Virus

To examine the tendency of mIBS025 to revert back to virulence, sequential passaging of the virus (up to 15 passages) in 10-day-old SPF embryonated eggs was performed. After every passage, presence of the virus was confirmed by spot HA test and HA titration before the next passage. Subsequently, RNA was extracted from allantoic fluids infected with P1, P3, P5, P10, and P15 for RT-PCR and sequencing of the region containing the F cleavage site. Results showed that the nucleotide and amino acid composition of mIBS025 F cleavage site remained unchanged throughout the 15 consecutive passages in SPF embryonated eggs. Furthermore, in vivo evaluation of the pathogenicity of P1, P3, and P5 egg passages revealed ICPI values within the range known for classical attenuated strains (Table 3). This clearly indicates that mIBS025 is genetically and phenotypically stable after multiple passaging in SPF embryonated eggs.

To further evaluate the stability of the recombinant virus, mIBS025 was sequentially passaged in the respiratory organs of 1-day old SPF chicks. During each passage, the chicks were observed for clinical signs and mortality for a period of 5 days, after which they were humanely sacrificed to recover their respiratory organs for virus detection using RT-PCR. The homogenized organs were then used to inoculate another set of chicks for the next passage until passage 12. Results indicate that inoculated chicks remained apparently healthy with no evidence of clinical signs following inoculation with various passages of the virus (up to P12). In addition, virus was detected in the respiratory organs of all the inoculated chicks throughout the experimental period. More importantly, the F cleavage site of the virus at various passages remained monobasic throughout the passaging period. This also indicates the stability of the virus after in vivo passaging in SPF chicks. 

### 3.5. Immunogenicity of NDV Strain mIBS025 and LaSota Vaccines

**Figure 3:**
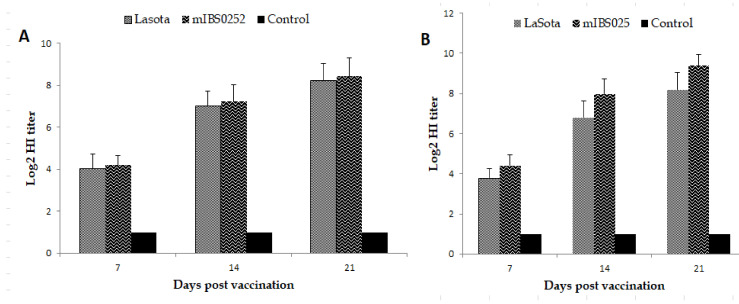
Post vaccinal HI antibody titers in 1-day old SPF chicks immunized with LaSota and mIBS025. Serum samples collected from vaccinated chicks (10 per group) at indicated time points post vaccination were subjected to HI test using (**A**) Lasota (genotype II NDV).and (**B**) wild type IBS025/13 (genotype VII NDV) as HA antigens. Results obtained were expressed as geometric mean titers (Log2) ± SD.

Serum samples collected at days 0, 7, 14, and 21 after vaccination in various groups were analyzed by HI tests using LaSota and IBS025/13 strains of NDV as HA antigens. Results obtained from the assay were expressed as geometric mean titers. Pre-vaccination HI titer in both the vaccinated and control groups was shown to be less than log2. Similarly, birds in the control group that were ‘mock vaccinated’ using PBS did not show any positivity to HI antibodies up to day 21 following vaccination. On the other hand, within one week after vaccination, there was a sharp rise in the HI titer of all the birds vaccinated with either NDV LaSota strain (4 ± 0.71) or NDV mIBS025 (4.4 ± 0.45) using homologous HA antigens (Figure 3). In both groups, the mean log2 titer kept on increasing steadily reaching 8.2 ± 0.84 and 9.4 ± 0.55 at 21 dpv in the LaSota and mIBS025 vaccinated groups, respectively (Figure 3). Therefore, while both vaccines induced a strong antibody response, the humoral response induced by mIBS025 appeared to be slightly higher than that of LaSota (*p* > 0.05) at all the time points regardless of the HA antigen used in determining the HI titer (Figure 3).

### 3.6. Protective Efficacy of NDV mIBS025 and LaSota

In order to compare the protective efficacies of LaSota and mIBS025, vaccinated birds were challenged with a highly virulent NDV strain IBS002/11 at 21 days post-vaccination. Each bird was then monitored and scored daily according to the criteria described above. The results indicate that birds in the control group began to manifest typical clinical symptoms of ND three days post-challenge (dpc), with initial morbidity of 25% which quickly reached 100% at 4 dpc. Clinical symptoms manifested by birds at 4 dpc included inappetence, depression, prostration, and incoordination with evidence of paralysis observed in some birds. At 5 dpc, greenish diarrhea, sneezing, and respiratory rales were observed in all the surviving birds, in addition to the symptoms observed on day 4. The pattern of mortality in the control group was 10% at 4 dpc, 50% at 5 dpc (Table 4), and 100% at 6 dpc. On the other hand, apart from the inappetence recorded in a few birds within the first 3 days after challenge, all the birds vaccinated with LaSota or mIBS025 remained healthy throughout the experimental period. Furthermore, no mortality was recorded in both LaSota and mIBS025 groups throughout the observation period. This indicates that both LaSota and mIBS025 completely protected birds against clinical disease due to genotype VII NDV challenge. There was no significant difference in the protective efficacy between LaSota and NDV mIBS025 vaccines (Table 4).

### 3.7. Post Challenge Cloacal and Oropharyngeal Virus Shedding

To estimate the quantity of the virus shed from both the vaccinated and control birds, qPCR based absolute quantification of the viral mRNA was performed. Accordingly, standard curve was generated based on the 10-fold dilution of a known concentration of NDV IBS002/11 RNA. Cloacal and oro-pharyngeal swabs collected from birds at 3, 5, 7, 10, and 12 days post-challenge were used to estimate copy numbers of the shed virus based on the generated standard curve. At day 3 post-challenge (dpc), all the sampled birds were positive for virus shedding regardless of the group and route. However, at this time point, the mean virus shedding titer was significantly higher in the control group than in the two vaccinated groups (*p* < 0.05). At day 7 post-challenge, none of the birds in the control group was alive. Therefore, samples were only obtained from birds in the vaccinated groups. Interestingly, the two vaccinated groups demonstrated a reduction in both the number of birds shedding the virus and the mean quantity of the shed virus through the cloacal and oropharyngeal routes. At day 10 post-challenge, the number of birds shedding the virus in the mIBS025 group became significantly less than those in the LaSota vaccinated group (Table 5 and Table 6). By the 12th day post-challenge, none of the birds vaccinated with mIBS025 was shedding the virus either through the cloaca or oro-pharyngeal routes, while in the LaSota vaccinated group, 4/10 birds were still shedding the virus via the cloacal route (Table 5) and oropharyngeal route (Table 6). Noteworthy, at nearly all the time points, the mean cloacal and oropharyngeal virus copy numbers obtained from the mIBS025 vaccinated group was significantly lower than those obtained from the LaSota vaccinated group (*p* < 0.05).

## 4. Discussion

Conventional vaccines such as LaSota, have contributed immensely to the global control of ND. In all countries where the disease is endemic, these vaccines are extensively used in order to reduce the substantial economic losses incurred annually due to the disease [38,39]. However, their protective efficacy in chicken is considerably threatened by several environmental, host-related, and vaccine-related factors. Of striking importance among these factors is the genotype mismatch between the vaccine strains and the circulating field strains, which has been attributed to the continuous emergence of NDV variants in different parts of the world [16]. LaSota and indeed most of the commercially available ND vaccines are derived from genotype II isolates. Whereas the most predominantly circulating NDV strains particularly in Southeast Asia belong to genotype VII. It has severally been shown that homologous vaccines that are genetically closer to the challenge strains (genotype-matched vaccines) have a better protective efficacy compared to the heterologous vaccines [31,40]. Consequently, for improved control of ND in different geographic locations, attention has now been shifted to the generation of genotype-matched vaccines.

Outbreaks of ND among vaccinated farms are a common occurrence in Malaysia [29,31,33] and indeed in the entire Asian continent [41,42]. Between 2004–2013, numerous NDV strains belonging to genotype VII were isolated from vaccinated farms in different parts of Malaysia. Among those isolates is NDV IBS025/13, which seems to be a naturally occurring recombinant strain between the vaccine and field strains of NDV. The entire viral NP and about two-thirds of its P genes were those of genotype II while the M, F, HN, and L were all closely related to those of genotype VII isolates [35]. More so, the genomic length of the isolate is 15,186 bp consistent with the so-called ‘early NDV genotypes’ isolated before the 1960s. Importantly, the virus is highly virulent and grows to a high titre in chicken embryonated eggs. These unique attributes of the virus collectively make it an excellent candidate for vaccine development. Therefore, in the present study, NDV IBS025/13 was rationally attenuated using reverse genetics and the attenuated virus was evaluated for improved protective efficacy against genotype VII NDV challenge in SPF chicken.

The F cleavage site has previously been shown to be the most important determinant of NDV virulence [11,43,44]. Therefore, we used reverse genetics to construct the antigenomic clone of NDV IBS025/13 with F cleavage site modified from polybasic to monobasic, as contained in all attenuated strains of NDV. A successful recovery of the attenuated version of the virus (mIBS025) was accomplished following the co-transfection of the full antigenomic clone with the helper plasmids. To ensure the stability of the genetic manipulation, mIBS025 was sequentially passaged 15 times in chicken embryonated eggs (in vitro stability) and infected allantoic fluids of P1, P3, P5, P10, and P15 were used to extract viral RNA for RT-PCR and determination of the stability of monobasic F cleavage site. Pathogenicity of P1, P3, and P5 infected allantoic fluids was also evaluated using ICPI. Our findings revealed that the virus not only remained attenuated but also retained its engineered monobasic F cleavage site throughout the period of passage, indicating the stability of the virus after repeated passage in chicken embryonated eggs. We further evaluated the in vivo stability of mIBS025 by passaging the virus in one-day old chicks up to 12 passages followed by RT-PCR and sequencing of the partial F gene containing the F cleavage site. Results indicate that the virus not only efficiently replicated in the respiratory organs of the infected birds but also retained the monobasic F cleavage site up to P12. This provides additional evidence that F cleavage site modification alone is enough to stably attenuate virulent genotype VII NDV as earlier reported by [45]. To our knowledge, this is the first application of reverse genetics to develop a recombinant genotype-matched live attenuated vaccine based on the Malaysian NDV genotype VII isolate. The previous study on genetic manipulation of another Malaysian isolate (genotype VIII AF2240-I strain) was for improved oncolytic efficacy on human cancer and therefore did not involve virus attenuation by F cleavage site modification [46].

The hemmagglutination inhibition (HI) test is the most popular assay in ND serology as it correlates very well with disease protection [47]. In this study, the test was utilized to assess the immunogenicity of conventional LaSota and the newly generated mIBS025 vaccines in SPF chickens using both homologous and heterologous HA antigens. Noteworthy, regardless of the HA antigen used, birds vaccinated with either LaSota or the recombinant vaccine showed a steadily increasing pattern of antibody titers from 0–21 days post-vaccination (dpv). On the other hand, the antibody titers from the control group remained lower than log2 up to 21 dpv. Indeed, at day 14, the antibody titer for all the birds in the LaSota and mIBS025 groups was above the protective threshold of 2^5^, signifying the high immunogenicity of the two vaccines. Undoubtedly, humoral immunity plays a pivotal role in protection against mortality, morbidity, and even reduction in virus shedding following virulent ND challenge [48,49]. In fact, a high HI titer has been strongly correlated with effective ND control [50]. Interestingly, despite administering the same doses of the vaccines, at all the time points (7, 14, and 21 dpv) the mean antibody titers for NDV mIBS025 vaccinated group was higher than those from LaSota vaccinated group irrespective of the HA antigen used in the assay. Furthermore, higher HI titers were generally recorded in both vaccinated groups when homologous HA antigen was used in the HI test than when heterologous HA antigens were used to measure the HI antibody titers. For instance, in the mIBS025 vaccinated group at 21 dpv, the mean HI antibody titer changed from 8.4 to 9.2 log2 when LaSota antigen (heterologous antigen) was replaced with (IBS025/13 HA antigen) in the HI test. Similar observations were made by Xiao et al. [41] who reported a log2 or in some cases 2 log2 fold difference when LaSota vaccinated birds were tested against genotype II or genotype VII HA antigens. At the moment, the developed vaccine was not designed for differentiating infected from vaccinated animals (DIVA) because of the similarity in the antibody profiles of the vaccine and other prevalent strains. Further improvement of the vaccine and the development of accompanying serological assay, may ensure the implementation of ND DIVA strategy. In addition, most poultry farms that used genotype VII NDV vaccine are also using LaSota vaccine to control ND outbreaks which further complicates the development of ND DIVA strategy.

In order to assess the protective efficacy of the vaccines, all the birds in both the vaccinated and non-vaccinated groups were challenged with a highly virulent NDV strain IBS002/11 via the occulo-nasal route. This challenge virus shares the same taxonomic group (genotype VII) with the wild type NDV strain IBS025/13 [35]. Protection against the challenge was measured by assessing the morbidity and mortality post-challenge among all the birds according to the recommendations of [36]. As expected, there was a 100% mortality and morbidity among the unvaccinated control birds, indicating their full susceptibility to the virulent challenge. Prior to their death, the birds began to manifest clear signs of the disease around 3 days post-challenge, with the clinical symptoms reaching a peak severity at 4–5 dpc, in agreement with the earlier report by [51] using different NDV isolates. Overall, the birds in the control group have a significantly higher morbidity score than the remaining vaccinated groups (*p* < 0.05) which remained healthy throughout the observation period post-challenge. This is a clear indication that the two vaccines are capable of preventing chicken from clinical disease and mortality as previously reported by [52].

Several studies have shown that ND vaccines may protect against overt clinical disease and mortality but not virus shedding [31,53]. The amount of the virus shed by the vaccinated birds is dependent upon the extent of vaccine-induced immunity, the virulence, and the genetic make-up of the challenge strain as well as the time frame between vaccination and challenge [50]. If the load of the virus shed from vaccinated birds is high and the shedding occurs over a long period of time, the outbreak of the disease could occur among the nearby unprotected birds which might either get the virus directly from the challenged birds or indirectly through fomites. Thus, an ideal vaccine should in addition to being highly immunogenic, be able to block or substantially reduce virus shedding post-challenge. Although virus shedding was observed in all the challenged groups in our study, birds vaccinated with NDV mIBS025 recorded the least cloacal and oropharyngeal virus shedding at almost all the time points (Table 5 and Table 6). This signifies that the newly generated recombinant vaccine is more effective in reducing both the number of birds shedding the virus as well as the mean quantity of the virus shed by the challenged birds. Importantly, NDV mIBS025 was derived from NDV IBS025/13 strain which belongs to the same genetic group (genotype VII) with the challenge virus (NDV IBS002/11) [35]. On the other hand, LaSota vaccine belongs to genotype II group which is evolutionarily distinct from the genotype VII [21]. Thus, the difference in the magnitude of the virus shedding observed between the two vaccines might be indicative of the role of genetic relatedness between the challenge and vaccine strains in determining the protective efficacy of NDV vaccines. Similar speculations were earlier made by Miller et al. [54].

## 5. Conclusions

Taken together, the prospects of reverse genetics technology in modern vaccine design have been explored in this study. Specifically, a genotype- matched live attenuated vaccine candidate was generated by manipulating the genome of a recently isolated naturally recombinant virulent genotype VII NDV isolate, at a motif in the F gene that controls the pathogenicity of the virus. Biological characterization of the generated recombinant virus revealed its complete loss of virulence as determined by the OIE recommended pathogenicity testing indices. Importantly, the newly acquired phenotype was found to remain unchanged after several passages in the respiratory organs of young chicks and in SPF chicken embryonated eggs, indicating the stability of the recombinant virus. Furthermore, immunization of SPF chicken with the vaccine candidate leads to the induction of a strong antibody-mediated immunity capable of fully protecting chicken against the virulent genotype VII challenge. In addition, a significantly reduced virus shedding was observed among the birds vaccinated with the vaccine compared to the unvaccinated birds or those vaccinated with LaSota vaccine. Therefore, the NDV mIBS025 generated in this study is a promising vaccine candidate for improved control of ND in Malaysia and neighboring countries.

## Figures and Tables

**Table 1:** Fusion protein nucleotide and amino acid distances between LaSota vaccine (JF950510.1) and other virulent NDV isolates in Southeast Asia.

Strain	Accession Number	Genotype	Nucleotide Divergence (%)	Amino Acid Divergence (%)	Year of Isolation	Country of Origin
IBS002	KR074404.1	VII	18.71	10.27	2011	Malaysia
IBS005	KR074405.1	VII	18.71	10.27	2011	Malaysia
MB076	KR074406.1	VII	17.07	10.18	2005	Malaysia
MB128	KR074407.1	VII	17.07	10.18	2004	Malaysia
IBS025/13	KT355595.1	VII	18.50	11.01	2013	Malaysia
NCXCP	MG869266.1	XII	19.42	12.62	2011	Vietnam
NDV15A1	MG869268.1	XII	19.10	10.71	2015	Vietnam
NDVLC15	MG869269.1	XII	18.83	11.35	2015	Vietnam
NDVQG	MG869270.1	XII	18.28	11.35	2008	Vietnam
NCXKH	MG869271.1	XII	18.60	11.93	2011	Vietnam
Ban 010	HQ697254.1	VII	18.47	11.06	2010	Indonesia
Suk 019	HQ697255.1	VII	18.45	10.46	2010	Indonesia
Muk 003	HQ697256.1	VII	18.31	10.46	2009	Indonesia
Sragen 014	HQ697258.1	VII	18.39	11.04	2010	Indonesia
Kud 018	HQ697260.1	VII	18.39	11.04	2010	Indonesia
Bali 020	HQ697261.1	VII	18.40	10.73	2010	Indonesia
Cockatoo 87	KF767104.1	VII	17.05	11.14	1988	Indonesia
Lory 88	KF767105.1	VII	17.45	10.81	1988	Indonesia
C300	KF767106.1	VII	17.26	10.62	1976	Indonesia
Belitung	MK069428.1	VII	19.18	11.23	2015	Indonesia
Kulonprogo	MK069429.1	VII	19.33	11.60	2017	Indonesia

**Table 2:** Biological features of the parental and rescued viruses.

Virus	Pathogenicity			Virus Titration	
	ICPI	MDT (Hours)	F Cleavage Site	EID_50_	HA Titre
IBS025/13	1.86	58.4	^112^RRQKRF^117^	9.2 log10	10 log2
mIBS025	0.00	150.4	^112^GRQGRL^117^	8.9 log10	8 log2

ICPI = Intracerebral pathogenicity index in 1 day old chicks, MDT = Mean death time of chicken embryonated eggs, F = Fusion protein, HA = hemagglutination.

**Table 3:** Stability of monobasic fusion protein cleavage site and lentogenic phenotype of NDV mIBS025 in SPF eggs and chicks.

Stability in SPF Embryonated Eggs				Stability in SPF 1-Day Old Chicks			
**Passage**	ICPI	HA Titre	F Cleavage Site	Passage	RT-PCR	Clinical Signs	F Cleavage Site
P1	0.00	8 log2	^112^GRQGRL^117^	P1	+	NIL	^112^GRQGRL^117^
P3	0.13	9 log2	^112^GRQGRL^117^	P5	+	NIL	^112^GRQGRL^117^
P5	0.00	8 log2	^112^GRQGRL^117^	P7	+	NIL	^112^GRQGRL^117^
P10	-	9 log2	^112^GRQGRL^117^	P10	+	NIL	^112^GRQGRL^117^
P15	-	8 log2	^112^GRQGRL^117^	P12	+	NIL	^112^GRQGRL^117^

ICPI = Intracerebral pathogenicity index (lentogenic strains have values of 0.0–0.7), HA = Hemagglutination, RT-PCR = Reverse transcriptase polymerase chain reaction, NIL = No clinical sign observed.

**Table 4:** Daily morbidity scores post challenge in vaccinated and control groups.

Days Post Challenge	PBS Control	LaSota Vaccine	mIBS025 Vaccine
1	0.0 ± 0.0	0.0 ± 0.0	0.0 ± 0.0
2	0.43 ± 0.53	0.14 ± 0.38	0.29 ± 0.49
3	1.14 ± 0.38	0.57 ± 0.53	0.43 ± 0.53
4	2.5 ± 0.54	0.29 ± 0.48 *	0.43 ± 0.53 *
5	2.67 ± 0.57	0.0 ± 0.0 *	0.0 ± 0.0 *
6–14	Nil	0.0 ± 0.0	0.0 ± 0.0

Values with * differed significantly with the control. Nil = no animal available for testing.

**Table 5:** Cloacal virus shedding among vaccinated and non-vaccinated birds at different time points post challenge.

Time Points	LaSota Vaccine			mIBS025 Vaccine		Control	
	Number of Positive/Total	Virus Copy Number Mean (log10) ± SD	Number of Positive/Total		Virus Copy Number Mean (log 10) ± SD	Number of Positive/Total	Virus Copy Number Mean (Log 10) ± SD
**3 dpc**	10/10	7.38 ± 0.36 *	10/10		6.17 ± 0.40 *	10/10	10.09 ± 0.09
**5 dpc**	10/10	6.81 ± 0.56 *	9/10		5.82 ± 0.21 *^,a^	5/5	9.81 ± 0.62
**7 dpc**	8/10	5.53 ± 0.88	7/10		4.59 ± 0.43	NS	-
**10 dpc**	5/10	4.96 ± 0.61	2/10		2.23 ± 0.03 ^a^	NS	-
**12 dpc**	4/10	4.14 ± 0.62	0/10		ND	NS	-

Values with * differ significantly with control within the same time point while those with ^a^ differ significantly with the LaSota group. NS = no survived chickens. ND = No virus shedding detected.

**Table 6:** Oropharyngeal virus shedding among vaccinated and non-vaccinated birds at different time points post challenge.

Time Points	LaSota Vaccine		mIBS025 Vaccine		Control	
	Number of Positive/Total	Virus Copy Number Mean (log 10) ± SD	Number of Positive/Total	Virus Copy Number Mean (log 10) ± SD	Number of Positive/Total	Virus Copy Number Mean (log 10) ± SD
3 dpc	10/10	6.58 ± 0.48 *	10/10	6.06 ± 0.87 *	10/10	9.04 ± 0.96
5 dpc	10/10	7.02 ± 0.56	8/10	4.67 ± 0.85 ^a,^*	5/5	9.11 ± 0.05
7 dpc	7/10	6.19 ± 0.50 *	6/10	4.07 ± 0.53 ^a^	NS	-
10 dpc	5/10	4.92 ± 1.26	2/10	2.14 ± 0.25 ^a^	NS	-
12 dpc	4/10	3.55 ± 0.49	0/10	ND	NS	-

Values with * differ significantly with control within the same time point while those with ^a^ differ significantly with the LaSota group, NS = no survived chickens, ND = no virus shedding detected, dpc = days post challenge.

## Supplementary Materials

The following are available online at https://www.mdpi.com/2076-393X/8/2/270/s1, Figure S1: Verification of helper plasmid constructs by (I) colony PCR amplification of NP, P and L and (II) restriction endonuclease digestion of pCIneo-NP, pCIneo-P and pCIneo-L using EcoRI, MluI and NotI.
