# Supplementary material for: Development of an Effective and Stable Genotype-Matched Live Attenuated Newcastle Disease Virus Vaccine Based on a Novel Naturally Recombinant Malaysian Isolate Using Reverse Genetics
Source: Vaccines (Basel). 2020 Jun 2;8(2):270. doi: 10.3390/vaccines8020270 (PMC7349954; doi:10.3390/vaccines8020270)
Supplement: Supplementary file 1 [file vaccines-08-00270-s001.pdf]

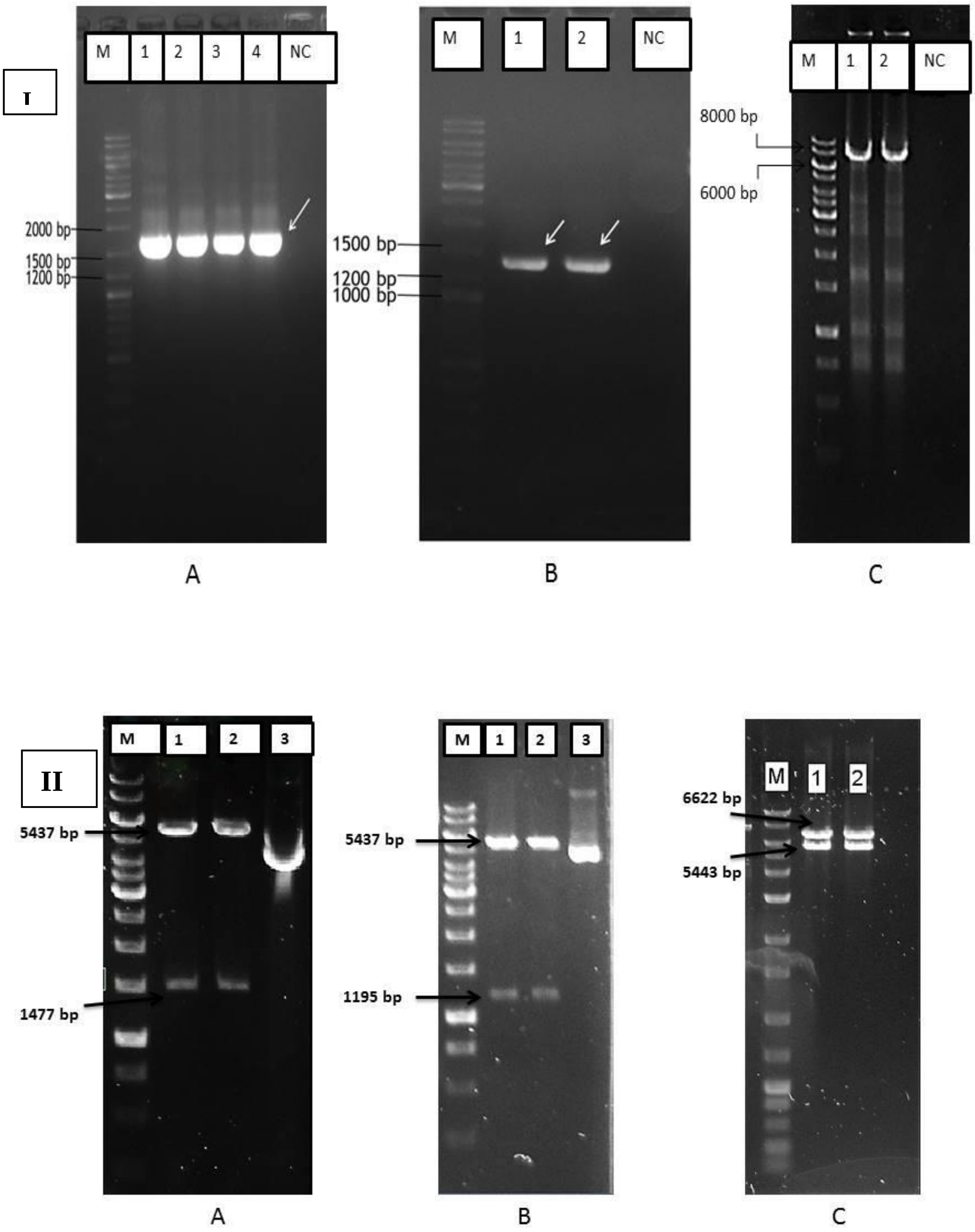

Figure S1. Verification of helper plasmid constructs by colony PCR and restriction analysis. IA, IB and IC respectively represent cloned NP coding region (1599 bp), Cloned f P gene (1317 bp) and Cloned L coding region (6750 bp) amplified using vector specific primers. IIA. Lanes 1 and 2 represent pCIneo-NP constructs digested with *Eco*RI and *Not*I. Lane 3 is the undigested plasmid construct. IIB Lanes 1 and 2 are pCIneo-P construct cut with *Eco*RI and *Not*I. Lane 3 is undigested pCIneo-P construct. (C) pCIneo-L constructs digested with *Mlu*I and *Not*I flanking the L coding region in the construct. M represents 1kb ladder.
